# Intra-Articular Giant Synovial Osteochondroma: Case Reports of the Ankle and Knee Joint

**DOI:** 10.1155/2015/320139

**Published:** 2015-02-16

**Authors:** Paolo Fornaciari, Pascal A. Schai, Richard Niehaus, Ulrich G. Exner

**Affiliations:** ^1^Clinic of Orthopedic Surgery, Fribourg Cantonal Hospital, Chemin des Pensionnats 2-6, 1708 Fribourg, Switzerland; ^2^Clinic of Orthopedic Surgery, Wolhusen Cantonal Hospital (LUKS), P.O. Box 365, 6110 Wolhusen, Switzerland; ^3^Clinic of General Surgery, Uri Cantonal Hospital, Spitalstrasse 1, 6460 Altdorf, Switzerland; ^4^Orthopedic Center Zurich (OZZ), Seestrasse 259, 8038 Zurich, Switzerland

## Abstract

Two cases of giant intra-articular osteochondromas (knee and ankle joint) are reported; pathologically they are rare representations of synovial chondromatosis. A 17-year-old man presented with a tumorous mass which had been localized in his left ankle for many years, increasing in volume during the last months. The lesion was removed by posteromedial ankle arthrotomy. The second case was observed in a 39-year-old woman with a slow-growing mass in her right knee joint. The lesion was removed from the Hoffa fat pad by open anteromedial arthrotomy.

## 1. Introduction

Synovial osteochondromatosis (SOC) is a benign lesion of nodular cartilaginous neoplastic development of the synovium that can lead to loose bodies within the articular space [1]. This condition is usually a monoarthritic disease and affects the knee joint in more than 50% of cases [2]. The disease occurs more commonly in men with a peak incidence in the fifth decade of life [3]. The main pathological characteristic is chondroid metaplasia of the subintimal tissue of synovial joints [4].

The term giant SOC was first used by Edeiken et al. [5] in 1994 to indicate synovial chondromas of more than 1 cm and occasionally reaching up to 20 cm of diameter. This giant form of SOC is rarely reported in literature, and various aspects of the condition are still unknown and it may represent a separate entity.

The patients described in this report presented with large intra-articular osteochondromatous lesions in unusual locations.

## 2. Case Report 1

**Figure 1:**
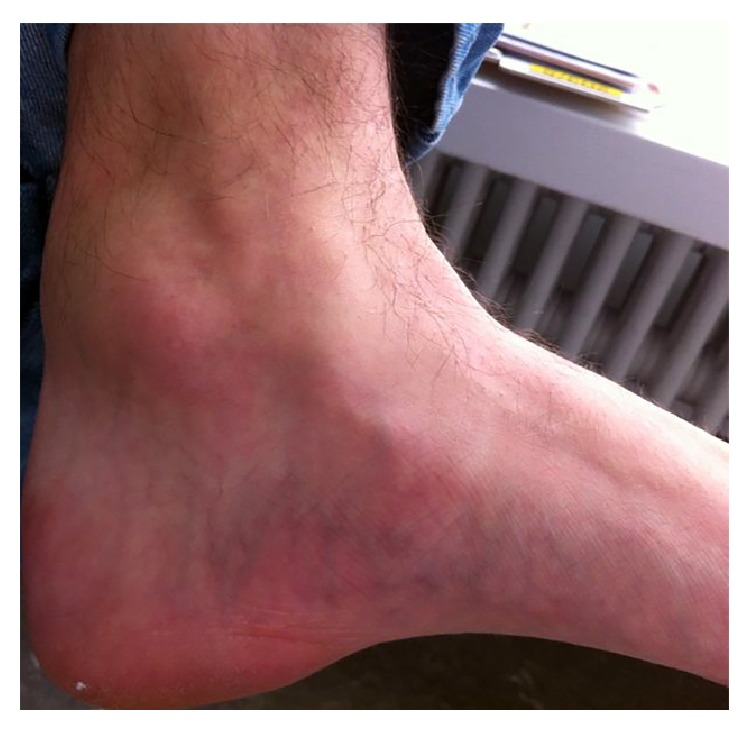
Clinical aspect of the tumorous mass adjacent to the ankle joint.

A 17-year-old male patient was referred to us because of a growing mass adjacent to the left posteromedial ankle (Figure 1). The lesion was noticed three years before and became symptomatic two months before presentation. This was due to compressive stress in the ankle by wearing ski- and inline skating-boots. The patient was otherwise healthy without any history of trauma. A solid 4 × 4 cm mass was detected by inspection and palpation of the posteromedial ankle. The plantar flexion and dorsal extension were both limited at 10°. The skin showed only slight redness; the ligament structures were stable; the neurovascular status was normal.

**Figure 2:**
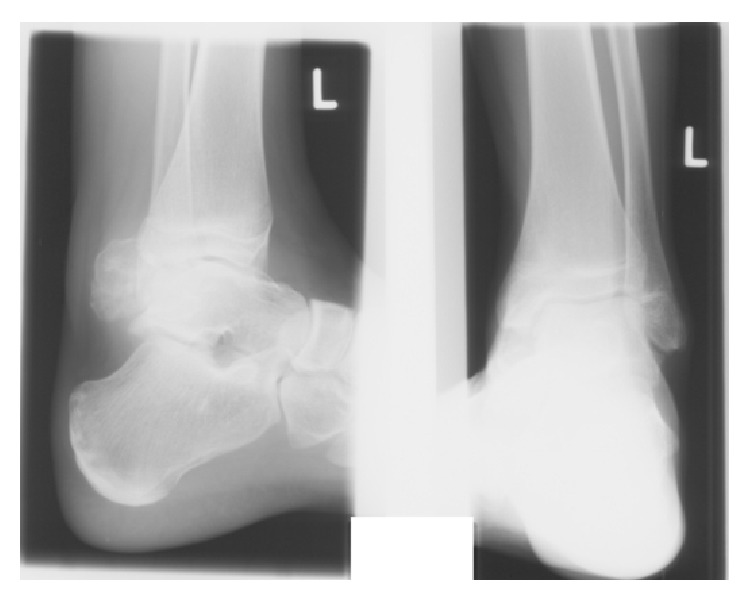
Plain radiography, anteroposterior and mediolateral view of the ankle joint.

**Figure 3:**
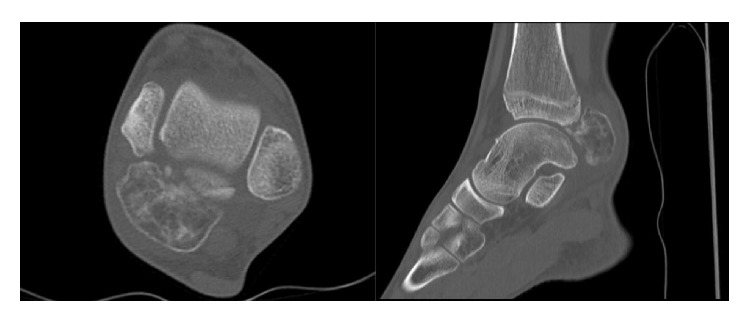
Preoperative CT images, axial and sagittal planes of the ankle joint.

Plain radiographs in anteroposterior and mediolateral projection of the ankle showed a polycyclic slightly condensed bone structure located dorsal to the tibiotalar joint (Figure 2). A CT scan of the ankle showed a large mass posterior to the talus with dimensions of 4.0 × 3.0 × 2.8 cm. Slight sclerotic reactions were visible. Similarly, discrete irregularities of the margins with focal osteolytic changes were found at the posteromedial talar contour. Mediocaudal to the large bone lesion, there was a second minor structure with soft tissue density of 2.8 × 1.7 × 1.2 cm. Otherwise the ankle joint was intact (Figure 3).

**Figure 4:**
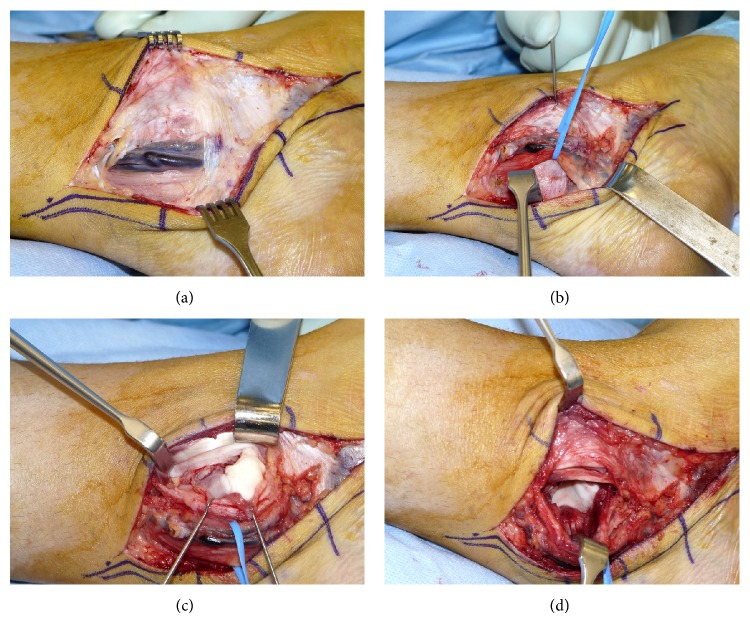
(a) Intraoperative aspect, neurovascular bundle (posterior tibial artery/veins, tibial nerve) and retinaculum of flexor muscles. (b) Intraoperative aspect, preparation and isolation of neurovascular bundle. (c) Intraoperative aspect, capsulotomy over the osteochondral lesion. (d) Intraoperative aspect, excision of the SOC tumor masses.

The patient underwent complete excision through a posteromedial arthrotomy of the tibiotalar joint (Figures 4(a)–4(d)). To obtain better access to the lesions and to preserve neighboring structures, an additional dorsal capsulotomy was performed. Two days postoperatively, after reabsorbing the swelling, early full-weight bearing and functional treatment were implemented.

**Figure 5:**
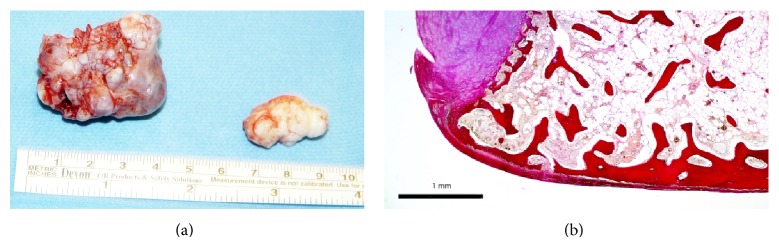
(a) Macroscopical aspect of the 2 masses from the ankle joint. (b) Corresponding microscopical aspect (Van Gieson's stain, magnification 2.5x).

The macroscopic aspects of the tumors were polylobular, plain delimited masses of bone and cartilage; the larger fragment was made of cancellous bone and cartilage of variable thickness (from 0.5 to 3 mm); the smaller one was completely composed of cartilage (Figure 5(a)).

Histological findings showed that the larger fragment was made of normal cancellous bone without increased osteoblastic or osteoclastic activity; there were normal adipocytes in the bone marrow; thin cartilaginous coverage was of normal appearance (Figure 5(b)). The smaller fragment was entirely of cartilage without pathological changes.

**Figure 6:**
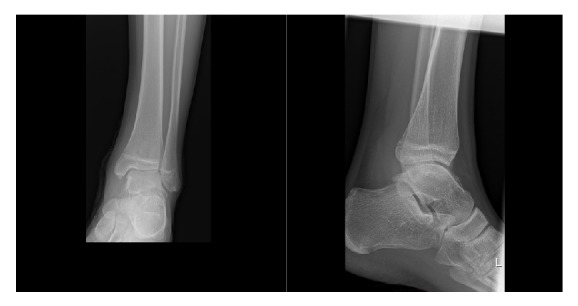
Plain radiography, anteroposterior and mediolateral view of the ankle joint at follow-up.

At the last follow-up appointment, two years after the operation, the patient reported no pain and no limitations during daily and recreational activities on any type of surface. Gait was normal with plantar flexion and dorsal extension in sagittal motion of 30° and 15°, respectively. The hindfoot alignment was normal. Function of collateral ligaments and neurovascular status were intact. Plain radiography did not show any relapse of the lesion (Figure 6).

## 3. Case Report 2

**Figure 7:**
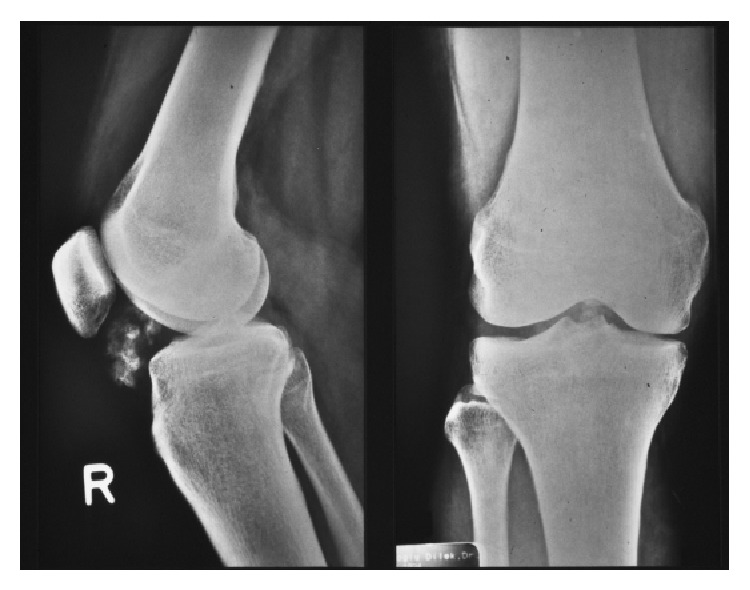
Plain radiography, anteroposterior and mediolateral view of the knee joint.

The patient, a 39-year-old woman in good health, presented with a prominent mass beside the patellar ligament in right knee joint. It was slow-growing and restricted flexion of right knee joint. Plain radiographs showed a partially calcified intra-articular lesion in the Hoffa fat pad (Figure 7).

**Figure 8:**
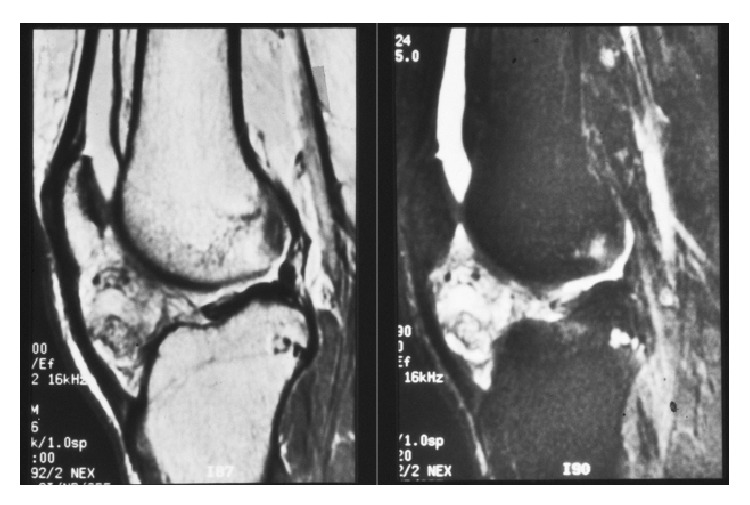
Preoperative MR images, sagittal plane T1- and T2-weighted images, of the knee joint.

**Figure 9:**
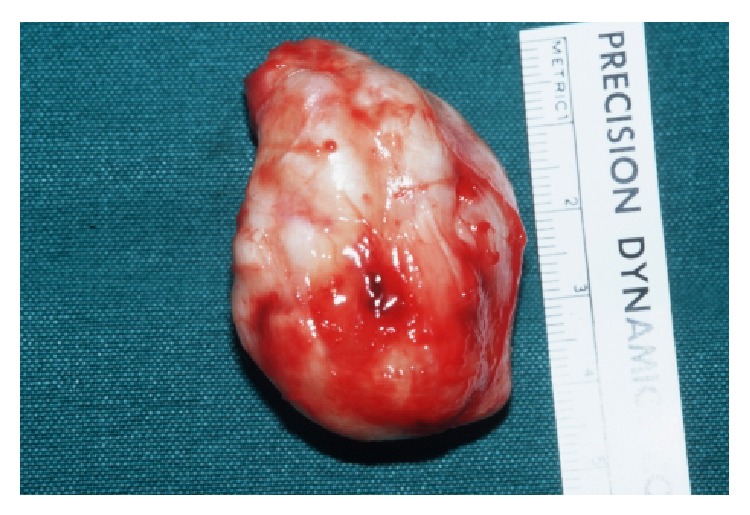
Macroscopic aspect of the tumorous mass in Hoffa's fat pad.

Exact location and structural analysis were confirmed with MR imaging. The masses were mostly of low signal intensity on T1-weighted MR images, but with some sites of high signal intensity corresponding to areas of calcification. In the T2-weighted MR images, the masses were heterogeneously of high signal intensity (Figure 8). The image pattern suggested a SOC of Hoffa's fat pad. The patient underwent an open arthrotomy with resection of the mass. The specimen with a maximum diameter of 5 cm was examined pathologically (Figure 9).

The mass was enclosed within Hoffa's fat pad in close contact to the synovialis.

Macroscopic aspects of the tumors were multiple nodules of which the largest one measured 5 × 3.8 × 2.5 cm, superficially lined with cartilage, with yellow bone tissue at the cut surface.

Histological findings were as follows: the largest lesion was described as cartilage with an uneven distribution of chondrocytes and with multiple foci of enchondral ossification. Peripherally, it was possible to sharply define a margin with the connective tissue of the synovial membrane. In the sample of joint capsule, no metaplastic cartilaginous foci were found.

At the clinical follow-up, 20 years after the operation, the patient reported no pain and no limitations during daily and recreational activities. Gait was normal with flexion and extension in sagittal motion of the knee of 120° and 0°, respectively.

## 4. Discussion

Giant SOC is rarely reported in the literature and its characteristics are largely unknown. Currently, our knowledge of giant form is chiefly based on that of the usual form of SOC.

The SOC diagnosis is based on concurrent clinical, radiological, and histological findings and exclusion of other conditions.

Clinical presentation of SOC is often subtle, with slow progression. Originating from articular synovial tissue, local tenderness on palpation, reduced range of joint motion, and palpable masses are frequently found [1].

The typical characteristics of “ring-and-arc” chondroid mineralization and bony erosions in the MR images are suggestive of synovial chondromatosis [6]. Computer tomography (CT) clearly depicts calcified bodies and allows better visualization of bone erosion, which is present in 20–50% of cases [7]. Magnetic resonance findings are more variable than CT, but the typical pattern (77% of cases) shows low to intermediate signal intensity with T1-weighted images and very high signal intensity with T2-weighted images with hypointense calcifications [7].

The primary pathological abnormalities are subsynovial cartilage neoplasia, synovial hyperplasia, and the production of round cartilaginous nodules, known as chondromas. These nodules may continue to grow, nourished by synovial fluid; most chondromas calcify and are then termed osteochondromas. These isolated osseous bodies will only continue to grow if they reattach to the synovium [8].

Milgram [9] suggested a three-phase evolution of SOC. They are described as Phase I, active intrasynovial disease with no loose bodies; Phase II, active intrasynovial pathologic tissue mixed with loose bodies; and Phase III, without synovial disease but with multiple free osteochondral bodies. During phase III, synovectomy would not be recommended. Since these pathological variations exist, not all authors believe that the majority of cases progress in any predictable pattern [7].

Differential diagnoses include chronic articular infection, osteoarthritis, pigmented villonodular synovitis, monoarticular inflammatory arthritis, and periarticular neoplasms such as synovial sarcoma [2]. SOC may occur secondary to trauma, avascular necrosis, osteoarthritis, rheumatoid arthritis, and osteochondritis dissecans [10].

With a reported relative risk of malignant transformation of 5%, synovial chondrosarcoma is a decisive differential diagnosis of high prognostic importance for the patient [11]. Shearer et al. in 2007 [12] stated that a distinction between these two entities may be difficult because of similarity of clinical and radiographic features. Clinical appearance, radiographic or advanced imaging, and histological evidence were recommended to be considered collectively to arrive at an accurate diagnosis.

Synovial chondrosarcoma typically presents irregular contours, clumping calcifications, and bony destructions. Permeative and destructive margins rather than an erosive margin with adjacent marrow invasion suggest a malignancy [1, 13].

The treatment of choice for SOC is surgical excision with an open or arthroscopical approach [8]. According to the analysis by Maurice et al. [14] the recurrence rate is 11.5%. Two surgical techniques are suggested, the first being excision of the nodules only. The second is the removal associated with extensive synovectomy. Synovectomy does not guarantee success, as reported by Church et al. [15].

The usual form of SOC in the ankle joint is a rarity. It was found in less than 5% of the cases [16]. To our knowledge, 15 studies with complete description of intra-articular SOC of the ankle [1, 12, 16–28] in a total of 21 patients are present in English-language literature. Among these studies, only that of Wagner et al. [21] reports a giant form of SOC.

While the literature describes the knee as the most common location of the usual form of SOC, the confined location in Hoffa fat pad is a rarity. Referring to the giant form, the study by Osti et al. [29] is to our knowledge the first and only other case of primary giant SOC confined in Hoffa's fat pad. This is comparable to our cases in terms of location and size, but recurrence was observed in that study three years after removal.

Because of the characteristic intra-articular localization of such lesions, the only chief complaint may be a reduced range of joint motion. In these terms, the two presented cases seem to be instructive. Surgery in the two presented cases of giant SOC showed lasting results, in the first case for a medium-term follow-up period (2 years) and in the second case for a long-term follow-up period (20 years). Given the pathological findings in presented cases, it remains unclear whether giant synovial chondromas are a distinct entity or a rare variant of the typical synovial chondromatosis with multiple small nodules.

Further reports and analyses of the giant form of SOC are necessary to improve our understanding of this pathological entity and its differences from the usual form to optimize clinical management.
